# Recent Progress of Fabrication of Cell Scaffold by Electrospinning Technique for Articular Cartilage Tissue Engineering

**DOI:** 10.1155/2018/1953636

**Published:** 2018-03-25

**Authors:** Yingge Zhou, Joanna Chyu, Mimi Zumwalt

**Affiliations:** ^1^Department of Industrial, Manufacturing, and System Engineering, Texas Tech University, Lubbock, TX, USA; ^2^Department of Orthopedic Surgery and Rehabilitation, Texas Tech University Health Sciences Center, Lubbock, TX, USA

## Abstract

As a versatile nanofiber manufacturing technique, electrospinning has been widely employed for the fabrication of tissue engineering scaffolds. Since the structure of natural extracellular matrices varies substantially in different tissues, there has been growing awareness of the fact that the hierarchical 3D structure of scaffolds may affect intercellular interactions, material transportation, fluid flow, environmental stimulation, and so forth. Physical blending of the synthetic and natural polymers to form composite materials better mimics the composition and mechanical properties of natural tissues. Scaffolds with element gradient, such as growth factor gradient, have demonstrated good potentials to promote heterogeneous cell growth and differentiation. Compared to 2D scaffolds with limited thicknesses, 3D scaffolds have superior cell differentiation and development rate. The objective of this review paper is to review and discuss the recent trends of electrospinning strategies for cartilage tissue engineering, particularly the biomimetic, gradient, and 3D scaffolds, along with future prospects of potential clinical applications.

## 1. Introduction

Tissue engineering has emerged as an alternative cell-based approach designed to substitute damaged organs with tissues generated* in vitro*. It overcomes the barriers of conventional allograft transplantation, such as the scarcity of donor organs, the complexity of surgery, and complicated postoperative care [1]. In many cases, it involves the utilization of a scaffold, an engineered porous supporting material for tissue regeneration. Scaffolds mimic the extracellular matrix (ECM) of the original tissues and reproduce or replicate the natural tissue environment. In addition, scaffolds can foster certain mechanical and biological properties to modify the behavior of different cell phases [2–4].

Articular cartilage is tissue located between bones, which can withstand compressive and shear forces several times that of human body weight for years due to its low friction and high load bearing capacity [5, 6], attributed to the unique composition of its ECM [7]. However, it is also the unique ECM that makes the repair of articular cartilage extremely difficult [8]. The micro fissures caused by repetitive cyclic loading might not be visible to the eye but continuously undermine the integrity of collagen fibrils and proteoglycan network [9], causing cartilage destruction over time and leading to degenerative joint disease or osteoarthritis. Osteoarthritis is no longer common only among the elderly but has extended to the younger population who also need a long-term solution for their painful problem [10].

Currently, surgical treatments for knee osteoarthritis include arthroscopic microfracture, Osteoarticular Transfer System (OATS), and autologous chondrocyte implantation (ACI), as well as open procedures such as osteotomy and arthroplasty. Unfortunately, they all fail to provide healthy and vigorous articular cartilage in the long run. For example, osteotomy and arthroplasty can lead to articular exacerbation and impairment of neighboring bones [5]. Cartilage tissue engineering, on the other hand, may provide a potentially better resolution in the treatment of knee osteoarthritis. With scaffolds manufactured especially for articular cartilage, the functional complexity of electrospun scaffolds provides significant advantages over other techniques such as allowing the mesenchymal stem cells to grow in a way that facilitates the formation of fibrous tissue structure. Electrospinning may be a viable approach to achieving highly aligned and biocompatible scaffolds to meet the demands for cartilage tissue engineering.

### 1.1. Electrospinning

**Figure 1:**
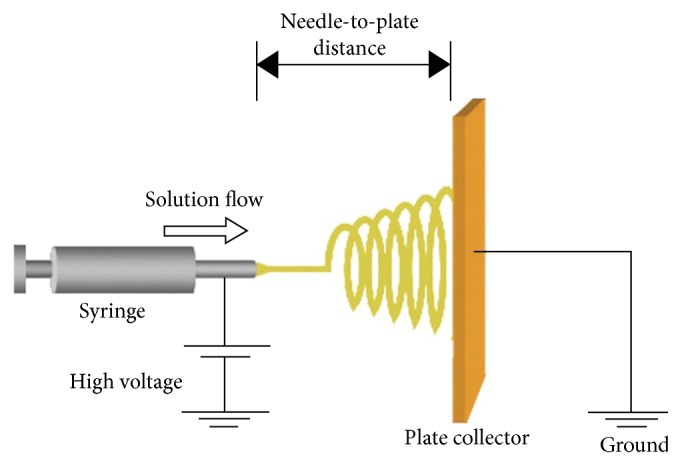
Typical electrospinning facility with a plate collector [6].

An electrospinning system is comprised of at least three units: a high voltage power unit, a material delivery unit (a capillary tube with a small spinneret in general), and a fiber collection unit. A typical electrospinning setup is shown in Figure 1 [6]. The randomly distributed nanofibers were collected in Figure 1 on a vertical plate. Usually, an advancement pump is employed to regulate the flow rate of polymeric solution discharged from a syringe. An electrically charged jet of polymer solution or melt is created by a high voltage source. The discharged polymer solution jet goes through an instability process, during which the jet is elongated and the solvent evaporates or solidifies. The jet is finally collected as an interconnected web of ultrathin fibers at the collector that is grounded or connected to an electrode. The diameters of electrospun fibers can range from 10 nm to 100 *μ*m depending on the material, device configuration, and process setup.

Electrospinning is able to generate loosely connected porous mats from submicrometer to nanometer scales in diameter as it is a highly flexible technique to produce continuous fibers. The nanofiber mat yields a high surface area to volume ratio and high porosity [54]. The nanostructure of the mat mimics the structure of the ECM in terms of both morphology and composition [55]. Various patterns of electrospun nanofibers have been fabricated for different biomedical applications ranging from artificial skin to endocrine organs and from the nervous system to cardiovascular tissues [56].

### 1.2. Electrospun Scaffolds for Cartilage Tissue Engineering

Electrospinning as a feasible and versatile technique can manipulate both natural and artificial polymers in nanoscale and promote the proliferation of mesenchymal stem cells (MSCs). Recent research attention has focused on improving the material design of the composition to produce biomimetic, gradient, and 3D scaffolds. Biomimetic scaffold is crucial in the reconstruction of connective tissue such as articular cartilage. Electrospinning may achieve the desired biomimetic characteristics with regard to biocompatibility and biomimicry by adding different synthetic and biological materials to the electrospinning nanofibers. Because tissue composition exhibits gradient features from the top layer that withstands high pressure to the bottom layer that connects with the subchondral bone, gradient scaffolds are needed for cartilage tissue engineering. Fibers generated during electrospinning tend to accumulate in a limited thickness and form a denser instead of thicker scaffold; therefore, strategies of fabricating thicker 3D electrospun scaffolds are crucial.

### 1.3. Objectives

The objective of this paper is to review and discuss the recent trends of electrospinning strategies for cartilage tissue engineering, particularly the biomimetic, gradient, and 3D scaffolds, along with future prospects of potential clinical applications in this field of research.

## 2. Method of Literature Search

Literature selection for this article was based on PubMed, Scopus, Google Scholar, ScienceDirect, and Web of Science databases from 2011 to the present, as the last review paper on this topic was published in 2012 [57]. The search was conducted using different combinations of the following terms: “tissue engineering,” “electrospinning,” “cartilage,” “scaffold,” and “bioscaffold.” Articles that were considered to be related to this review and written in English were included.

## 3. Literature Review

The current literature review reveals that the most prevailing recent trend is the fabrication of biomimetic, gradient, and 3D bioscaffolds, as presented below.

### 3.1. Biomimetic Composite Scaffolds

The desired characteristics of electrospun scaffold for cartilage regeneration include biocompatibility and biomimicry. Different synthetic and biological materials may be added to electrospinning nanofibers to achieve these characteristics. Cao et al. added graphene oxide with poly(vinyl alcohol) (PVA) and chitosan for better biocompatibility and produced uniform nanofibers with improved cell growth rate compared with nanofibers of chitosan and PVA alone [23]. Bioprinting and electrospinning were combined to fabricate biomimetic scaffolds for cartilage tissue engineering [13]. Electrospun fiber assembled hydrogel was also utilized for supplementation of glycosaminoglycan enriched and mineralized cartilage [18].

#### 3.1.1. Structure Biomimicry

Combination of natural and artificial polymers for scaffolds fabrication is considered promising for repair of cartilage defect or damage. For example, better chondrocytes adhesion and spreading efficiency were achieved with a poly(L-lactic-acid) (PLLA)/silk fibroin (SF) composite scaffold [20]. Poly(L-lactide-co-caprolactone) (P(LL-CL)) and gelatin were used for porous scaffolds fabrication to improve water absorption, cell infiltration, and shape-forming [21]. He et al. investigated the feasibility of collagen/poly(L-lactic acid-co-*ε*-caprolactone) membranes to facilitate cartilage-like tissue formation [14]. Similarly, Sadeghi et al. blended polyhydroxybutyrate (PHB) with chitosan (CTS) to produce hydrophilic fibrous scaffold for better chondrocytes attachment [22]. The advantages of gelatin/polycaprolactone (PCL) on the formation of 3D cartilage regeneration have also been demonstrated [17, 58].

Biocompatible polymers can be electrospun with wound healing and antimicrobial agents, such as alkanin and shikonin, to achieve high drug capturing efficiencies and multifunctional activities [11]. Yin et al. fabricated core-shell structure nanofibers with embedded kartogenin solution as core fluid to facilitate cartilage regeneration [24]. Mirzaei et al. incorporated glucosamine into PLLA/PEG scaffolds to achieve an enhanced cell proliferation rate [25]. These composite structures can be used to facilitate chondrogenic differentiation and cartilage repair. Oriented electrospinning scaffold thereby exhibits superb biomimicry of articular cartilage by modifying cell adhesion and distribution.

Posttreatment can also benefit biomimetic scaffold fabrication. Liu et al. freeze-dried electrospun fibers with a tricalcium phosphate and produced a trilayered scaffold consisting of oriented fibrous network providing promoted orientation of mesenchymal stem cells [15].

#### 3.1.2. Signal Biomimicry

Sustained and stable release of biomolecules such as growth factors, biological signals, and proteins remains an issue that needs to be resolved. Growth factors such as Nel-like molecule-1 have been preloaded in nanofibers via two-phase electrospinning to achieve prolonged release time as well as enhanced MSCs growth rate [26]. A core-sheath structure fibrous electrospun scaffold was fabricated by Man et al. Using a polyvinyl pyrrolidone/bovine serum albumin/rhTGF-*β*1 composite solution as the core fluid and poly(*ε*-caprolactone) solution as the sheath fluid, the scaffolds revealed sustained rhTGF-*β*1 release and promoted chondrogenic differentiation [16]. MSC chondrogenesis was also enhanced on cellulose derived glycosaminoglycan mimetic scaffolds by Huang et al., given that glycosaminoglycans provide signaling cues in native cartilage tissue [19]. Liao et al. coated cartilaginous ECM on electrospun PCL microfiber scaffolds to enhance MSC chondrogenesis [59]. Composite scaffolds such as poly(vinyl alcohol)/polycaprolactone (PVA/PCL) scaffold seeded with MSC also showed better cartilage defects healing effect [12].

Some important electrospinning-based methods to fabricate biomimetic composite scaffolds for cartilage tissue engineering are summarized in Table 1. All of the papers cited in Table 1 are directly or possibly related to articular cartilage tissue engineering applications, such as tracheal cartilage engineering.

### 3.2. Gradient Scaffolds

Several methods of generating gradient scaffolds have been reported by researchers such as structure and composition gradient. He et al. first deposited a layer of aligned nanofibers of pure poly(L-lactide-co-glycolide) acid (PLGA) on a cylindrical surface and then deposited a layer of aligned nanofibers of PLGA/nanohydroxyapatite (HA) on top. The microstructural organization and nano-HA content in electrospun scaffolds exhibit gradient change [33] and the anisotropic strain was also eliminated in scaffolds with fiber diameter gradient in Grey et al.'s research [30]. Guided spatial differentiation of MSCs [28] was also observed in composition and biomolecule gradient scaffolds. The scaffolds exhibit superior material properties in burst testing. The biodegradation rate and biological functions were controlled in Angarano et al.'s fiber diameter gradient scaffolds [31].

Fabricating scaffolds with gradients of molecules and element particles for tissue engineering has drawn substantial attention. Chondroitin sulfate (CS) and gentamycin sulfate (GS) gradients have been achieved with mineralized cartilage [18] and spatiotemporal drug release [35]. Ramalingam et al. reported a scaffold with amorphous calcium phosphate nanoparticles (nACP) gradient by coelectrospinning. Adhesion and proliferation of osteogenic cells were enhanced in gradient regions that have higher nACP concentration with a graded osteoblast response [29]. The graded cells' outgrowth rate and osteogenesis were also found to be positively related to the protein concentration gradient [27] and mineral gradient [34] on scaffolds, respectively.

Scaffolds with chemical and mechanical property gradient have also been investigated. Samavedi et al. demonstrated the possibility of fabricating a mechanochemical gradient scaffold with graded meshes by using controlled infusion of encapsulated CaCO_3_ and TiO_2_ nanoparticles [60]. The cell viability has shown preliminary evidence regarding the suitability of these scaffolds for* in vitro* tissue engineering.

Some important electrospinning-based methods to fabricate gradient scaffolds used in cartilage tissue engineering are summarized in Table 2. All of the papers cited in Table 2 are directly or possibly related to articular cartilage tissue engineering applications, such as interfacial tissue engineering and soft tissue regeneration.

### 3.3. 2D Scaffolds versus 3D Scaffolds

One of the major issues of conventional electrospun nanofibrous scaffolds is the limited thickness (typically < 1 mm) [61]. While 2D scaffolds are widely used in tissue engineering applications, they still fall short of contributing to crucial factors such as cell communication in ECM context, mechanical cues, and nutrient transportation [62]. 3D scaffolds, on the other hand, have superior cell differentiation and development rate, especially for connective tissues such as articular cartilage [53], and can improve cell infiltration rate with larger pore size and higher porosity [50]. The oriented electrospun fibers in 3D scale can guide the MSCs to grow in certain direction and further promote the formation of oriented and connective tissue. Further, in fabricating 3D multiphasic scaffolds where the scaffolds are separated spatially into different sections for guided MSCs differentiation mimicking the structure of connective tissues such as articular cartilage, electrospinning as a less complicated process for producing aligned fibers in 3D scale could demonstrate advantages in fabricating multiphasic scaffolds.

Among a multitude of applications for 3D organization of the nanofibers, processing the nanofibrous mesh into a desirable form after electrospinning has attracted substantial interest. For example, a 3D scaffold can be created by stacking multiple layers of nanofiber mesh for cell seeding [48]. Garrigues et al. found that multilayer scaffolds enhanced cell infiltration aggrecan (ACAN) gene expression [46]. Yunos et al. designed a bilayered scaffold by combining electrospun fibers with bioglass and obtained viable chondrocyte cells in the scaffold [43]. Xue et al. prepared the electrospun gelatin/PCL membranes into rounded shape with seeded chondrocytes to culture an ear-shaped cartilage, with good elasticity and impressive mechanical strength [45]. Kai et al. incorporated electrospun fibers into hydrogel to improve mechanical properties such as compressive strength [39]. The advantage of this simple method is the high degree of flexibility in creating the ultimate geometry of scaffolds.

Modified electrospinning setups can also be utilized for 3D structure such as electrospinning with external heat source [38] and dual nozzle extrusion system to improve cell infiltration rate. He et al. increased the pore size of scaffolds by near-field electrospinning [63]. Levorson et al. also increased cellular infiltration with multiscale scaffold, which contains microfibers and nanofibers evenly distributed throughout the entire construct [44]. Human mesenchymal cells were found to maintain scaffold cellularity under serum-free conditions with these scaffolds. A fluffy 3D scaffold with randomly and evenly oriented fibers in all directions was also fabricated by utilizing the electrostatic repulsion between fibers; the cell proliferation rate observed was 5 times as fast as the 2D scaffold counterparts after one week [42].

Novel electrospinning solutions have also been investigated for 3D electrospinning. For example, Xu et al. started from a polymer solution with de-cross-linked keratin from chicken feathers to obtain intrinsic water stability for 3D scaffold [47]. Enhancement of cartilage repairing can also be achieved by scaffolds with cross-linked hyaluronic acid [51] and biological cue chondroitin sulfate-incorporated electrospinning [37]. Wei et al. combined poly[(butylene succinate)-co-adipate] (PBSA) and PLLA to culture primary human chondrocytes (PHCs) in electrospun 3D fibrous scaffolds for improved cell attachment and proliferation [40].

Another strategy for fabricating 3D scaffold is combining electrospinning with other advanced manufacturing techniques such as 3D printing and freeze-drying. In the research by Xu et al. about hybrid inkjet printing and electrospinning system, the cell viability was 80% after one week, and cartilage-like tissue has formed both* in vitro* and* in vivo* [13]. Afonso et al. combined another advanced manufacturing technique, direct writing, with electrospinning to obtain oriented fibers and a highly controlled scaffold structure [52]. Liu et al. enhanced the mechanical strength of electrospinning by freeze-drying, and osteochondral defects were repaired in a rabbit model [49].

Last but not least, 3D structure can be achieved with different postprocess treatments. Holmes et al. improved mechanical properties of the 3D scaffolds with hydrogen treated multiwalled carbon nanotubes (MWCNTs) [41]. Zhu et al.'s scaffolds enhanced chondrogenic differentiation and cell infiltration rate with cold atmospheric plasma treated electrospinning [50]. Chen and Su also demonstrated better viability, proliferation, and differentiation of rabbit articular chondrocytes, with plasma-treatment electrospun scaffold [36].

Some important electrospinning-based methods to construct 3D nanostructures for the scaffolds used in tissue engineering are summarized in Table 3. All of the papers cited in Table 3 are directly or possibly related to articular cartilage tissue engineering applications, such as osteochondral tissue replacement and MSC chondrogenesis.

## 4. Discussion

Electrospinning provides an efficient way of fabricating cartilage tissue engineering scaffolds with nanoscale elements and utilization of a wide range of polymers. The process can be easily modified for various biomedical applications. Although there have been progress and improvements in recent years, very limited research has been reported on complete articular cartilage repair or major improvement in tissue engineering for comprehensive treatment of related diseases such as arthritis. There is hardly any parameter optimization for refined fiber strength and geometry. The complexity of electrospinning setups is growing rapidly as it incorporates more and more novel techniques such as biological cue-incorporated electrospinning [37], hydrogel [39], hybrid 3D printing [13], and cold atmospheric plasma treatment [50]. On one hand, it is highly challenging to deal with greater magnitude of parameters and characteristic architectures. On the other hand, these 3D scaffold strategies incorporating different techniques all come with certain weaknesses or vulnerabilities such as small thickness and microscale (instead of nanoscale) fibers (>200 nm). Therefore, the possibility of clinical application of these techniques on a large scale is still fairly slim.

Several electrospinning strategies have been developed for fabricating biomimetic composite scaffolds. Along with antimicrobial agents and growth factors, both structure and signal biomimetic scaffolds produced exhibit suitable biocompatibility and intermediate level of biomimicry. However, the effects of fiber-guided cell orientation on chondrogenic differentiation, attachment, and mineralization of marrow mesenchymal stem cells need to be further investigated and so should optimization for cell culture and electrospinning process.

Various improvements in mechanical properties of scaffolds, such as microstructural organization [33], tensile strength [60, 64], and anisotropic strain [30], have been achieved with different gradient properties. However, all of the improved mechanical properties, such as tensile strength, are still in the undesired low range (<10 MPa) [13]. And there are no quantitative results for the effect of enhanced mechanical properties on cell proliferation rate. In some cases, the overall mechanical strength was found to be even lower than that of the control scaffolds with no gradient. To address this issue, 3D electrospun fibers were immersed into a medium, such as hydrogel, to improve their mechanical strengths [39]; however, the electrospun fibers dissolve much faster than hydrogel in cell culture. Also, hardly any research has addressed the issue of mechanical properties of 3D scaffolds. These issues need to have experimental justification to verify that the improvement of these mechanical properties is helpful for* in vitro *and* in vivo* cell proliferation.

Using different gradient scaffolds, researchers were able to create graded cell response [29], outgrowth rate [27], and biodegradation rate [31] in a single scaffold.* In vivo* studies are needed to investigate the effects and suitability of these gradient scaffolds employed in cartilage implants, as well as related interfacial tissue engineering.

Although electrospun scaffolds possess higher porosity than other forms of scaffolds, a common drawback is the limited size of pores, which further prevents cells from growing in the scaffold [65]. Several measures have been investigated to address this issue, such as using sacrificial fibers [66], multiscale scaffold [44], hybrid scaffold [67], near-field electrospinning [63], and salt leaching and ice crystals [20]. These techniques could also be incorporated in 3D scaffolds fabrication for better cell infiltration.

The positive effects of 3D scaffolds on cell infiltration rate [44, 63] and cell proliferation rate [42] have been extensively investigated and verified. Other crucial properties in cell culture such as water stability [47] and cartilage-like tissue formation [37, 51] have also been investigated, demonstrating the intrinsic advantages of 3D scaffolds over 2D scaffolds. The biodegradation rate and biomimicry level should be further characterized quantitatively before 3D scaffolds can be implemented as cartilage implants* in vivo.*

Electrospun scaffolds for cartilage tissue engineering still face major challenges in biomechanical and biological properties. More novel synthetic and artificial materials need to be explored and tested with regard to issues such as mechanical strength, biotoxicity, biocompatibility, and biodegradability before research focus can switch to large-scale applications. Fabrication procedures need to be modified and improved in terms of facility and process parameters for better biomimicry and cost effectiveness. After further optimization of biomimetic, gradient, and 3D electrospun scaffolds along with the incorporation of advanced manufacturing techniques and novel materials, electrospun scaffolds exhibit substantial potential for cartilage tissue engineering.

## 5. Conclusion

This paper presents a variety of electrospinning strategies with novel materials and manufacturing techniques of bioscaffolds for cartilage tissue engineering. The literature review reveals that the most prevailing recent trend is the fabrication of biomimetic, gradient, and 3D bioscaffolds. Natural polymeric and biological materials have been incorporated into electrospinning technique with synthetic materials to improve biomimicry and biocompatibility. Multiple approaches have been taken in electrospinning to form 3D scaffolds with cartilage-like structure and enhanced chondrogenic differentiation. Mechanical properties of scaffolds have been improved by various electrospinning strategies to achieve acceptable levels. Future research should focus on issues such as mechanical strength, biotoxicity, and biodegradability* in vivo* to pave the way for possible human trials.

## Figures and Tables

**Table 1:** Summary of electrospinning-based techniques for fabrication of biomimetic composite scaffolds.

Authors	Year	Technique	Results	Application
Kontogiannopoulos et al. [11]	2011	Incorporated wound healing and antimicrobial agents	High drug entrapment efficiencies and multifunctional activities	Tissue engineering scaffolds

Shafiee et al. [12]	2011	Seed MSC in electrospun scaffold	Improved cartilage defects healing	Bone and cartilage tissue engineering

Xu et al. [13]	2012	Bioprinting and electrospinning	80% survived chondrocytes and cartilage-like tissue formation	Cartilage tissue engineering

He et al. [14]	2013	Electrospinning	Cartilage-like tissue formation	Cartilage tissue engineering

Liu et al. [15]	2014	Freeze-dried electrospinning	Successful regenerated osteochondral defects	Triphasic osteochondral implant

Man et al. [16]	2014	Electrospinning	Sustained rhTGF-*β*1 release and better chondrogenic differentiation	Cartilage tissue engineering

Zheng et al. [17]	2014	Electrospinning	3D cartilage regeneration	Cartilage tissue engineering

Mohan et al. [18]	2015	Electrospun fiber assembled hydrogel	Sustained release of chondroitin sulfate	Cartilage tissue engineering

Huang et al. [19]	2016	Electrospinning	Enhanced MSC chondrogenesis	Cartilage tissue engineering

Li et al. [20]	2016	Composite electrospinning	Better chondrocytes adhesion and filtration	Cartilage tissue engineering

Kalaithong et al. [21]	2016	Electrospinning and wet spinning	Better water absorption and cell infiltration	Cartilage tissue engineering

Sadeghi et al. [22]	2016	Electrospinning	Better hydrophilic property and cell attachment	Cartilage tissue engineering

Cao et al. [23]	2017	Chitosan/graphene oxide polymer nanofiber	Better biocompatibility and cell growth rate	Cartilage tissue engineering

Yin et al. [24]	2017	Core-shell structure nanofiber with embedded kartogenin solution	Promoted chondrogenic differentiation	Tracheal cartilage regeneration

Mirzaei et al. [25]	2017	Glucosamine incorporated into PLLA/PEG scaffolds	Enhanced cell proliferation rate	Cartilage tissue engineering

Wang et al. [26]	2017	Two-phase electrospinning	Prolonged drug release time and enhanced MSCs growth rate	Cartilage tissue engineering

**Table 2:** Summary of electrospinning-based techniques for fabricating gradient scaffolds.

Authors	Year	Gradient parameter(s)	Technique	Results	Application
Zander et al. [27]	2012	Protein concentration gradient	Air-plasma-modified electrospinning	Corresponding cell outgrowth rate	Tissue engineering

Zhang et al. [28]	2012	Composition and biomolecule gradient	Microfluidic assisted electrospinning	Guided spatial cell differentiation	Tissue engineering

Ramalingam et al. [29]	2013	Composition gradient	Coelectrospinning	Enhanced osteogenic cells proliferation and adhesion	Interfacial tissue engineering

Grey et al. [30]	2013	Fiber diameter gradient	Gradient electrospinning	Enhanced mechanical properties	Tissue engineering

Angarano et al. [31]	2013	Fiber diameter gradient	Reactive electrospinning	Gradient biodegradation rate	Soft tissue regeneration

Sundararaghavan et al. [32]	2013	Growth factor gradient	Gradient electrospinning	Directed cell motility in gradient direction	Tissue engineering

He et al. [33]	2014	Structure and composition gradient	Modified coelectrospinning	Gradient cell metabolic activity	Tissue engineering

Liu et al. [34]	2014	Mineral gradient	Graded mineral coating	Graded mesenchymal stem cells osteogenesis	Interfacial tissue engineering

Mohan et al. [18]	2015	CS and BG gradient	Electrospun fiber assembled hydrogel	Glycosaminoglycan enriched and mineralized cartilage formation	Cartilage tissue engineering

Liu et al. [35]	2016	GS and deferoxamine	3D bioprinting and electrospinning	Spatiotemporal drug release	Osteochondral tissue engineering

**Table 3:** Summary of electrospinning-based techniques for fabricating 3D nanofibrous structure.

Authors	Year	Technique	Advantage(s)	Disadvantage(s)	Application
Chen and Su [36]	2011	Electrospinning with plasma treatment	Enhanced chondrocytes viability and proliferation	N/A	Cartilage tissue engineering

Coburn et al. [37]	2012	Biological cue chondroitin sulfate incorporated electrospinning	Enhanced cartilaginous formation	Weak mechanical properties	Cartilage tissue engineering

Shabani et al. [38]	2012	Modified setup of electrospinning with heat from halogen light bulbs	Improved cell infiltration rate	Material limitation	Tissue engineering

Kai et al. [39]	2012	Nanofiber with hydrogel	Relatively higher compressive strength	No significant cell proliferation improvement	Tissue regeneration

Xu et al. [13]	2012	Hybrid inkjet printing/electrospinning system	High cell viability, formed cartilage-like structure	Further refinement required	Cartilage tissue engineering

Wei et al. [40]	2012	Electrospinning	Improved cell attachment and proliferation	N/A	Cartilage tissue engineering

Holmes et al. [41]	2013	Hydrogen treated multiwalled carbon nanotubes (MWCNTs)	Higher mechanical strength and cell differentiation	Unclear effect of MWCNTs *in vivo*	MSC chondrogenesis

Cai et al. [42]	2013	Electrostatic repulsion	Randomly and evenly oriented 3D fibers	Rapid delivery of electrons on fibers required	Cell culture for soft tissues

Yunos et al. [43]	2013	Bilayered scaffold	Chondrocyte cell-supporting ability	Decreased HA formation rate with thicker layer	Osteochondral tissue replacement

Levorson et al. [44]	2013	Dual extrusion electrospinning	Maintained scaffold cellularity	Lack of parameter optimization	Cartilage tissue engineering

Xue et al. [45]	2014	Prepare the electrospun membrane in rounded shape	Formed ear-shaped cartilage tissue	Lack of immunogenicity investigation	Cartilage tissue engineering

Garrigues et al. [46]	2014	Electrospinning	Enhanced cell infiltration	Lower elastic modulus	Cartilage tissue engineering

Xu et al. [47]	2014	Electrospinning solution with de-cross-linked keratin from chicken feathers	Intrinsic water stability	Randomly oriented fibers	Cell penetration and differentiation

Orr et al. [48]	2015	Vertical stacking layers of fiber membrane	Easy to seed cells on surface prior to stacking	Cells unable to penetrate through layers	Compressive loading applications

Liu et al. [49]	2015	Electrospinning and freeze drying	Better mechanical strength	N/A	Cartilage tissue engineering

Zhu et al. [50]	2015	Electrospinning with cold atmospheric plasma treatment	Enhanced chondrogenic differentiation and cell infiltration	Small thickness for 3D scaffold	Cartilage tissue engineering

Chen et al. [51]	2016	Modified scaffold with cross-linked hyaluronic acid	Superabsorbent property and excellent cytocompatibility	Complicated fabrication process	Cartilage tissue engineering

Afonso et al. [52]	2016	Direct writing electrospinning	Directed tissue organization and fibril matrix orientation	Microscale fibers	Tissue engineering

Damaraju et al. [53]	2017	Piezoelectric fibrous scaffolds	Promoted mesenchymal stem cell differentiation	N/A	Cartilage and bone tissue engineering
